# The effect of protein intake on athletic performance: a systematic review and meta-analysis

**DOI:** 10.3389/fnut.2024.1455728

**Published:** 2024-11-06

**Authors:** Shiao Zhao, Hong Zhang, Yipin Xu, Jiarui Li, Senyao Du, Ziheng Ning

**Affiliations:** ^1^Faculty of Health Sciences and Sports, Macao Polytechnic University, Macau, Macao SAR, China; ^2^Guangdong Provincial Key Laboratory of Human Sports Performance Science, Guangzhou Sport University, Guangzhou, China

**Keywords:** protein intake, athletic performance, macronutrients, physiological index, endurance ability, muscle strength

## Abstract

**Background:**

The impact of a protein-rich diet and protein supplements on athletic performance remains a topic of debate. Does protein intake offer benefits for athletes? If so, which specific aspects of athletic performance are most influenced by protein?

**Methods:**

This study aimed to explore the relationship between protein intake and athletic performance. A systematic database search was conducted to identify randomized controlled trials (RCTs) examining the effects of protein intake on athletes’ performance. The databases searched included PubMed, Scopus, Web of Science, EBSCO, and Ovid. The meta-analysis included a total of 28 studies involving 373 athletes. The meta-analysis employed both the fixed-effects model and the random-effects model to investigate the impact of protein intake on sports performance. Subgroup analyses were conducted to provide solid evidence to explain the results of the meta-analysis. Sensitive analysis and funnel plots were used to assess the risk of bias and data robustness.

**Results:**

Overall, protein intake did not show a statistically significant improvement in athletic performance (standardized mean difference [SMD] = 0.12, 95% confidence interval [CI]: −0.01 to 0.25). However, in subgroup analysis, the protein group demonstrated a statistically significant improvement in endurance performance, as indicated by the forest plot of final values (SMD = 0.17, 95% CI: 0.02 to 0.32). Additionally, the change value in the forest plot for endurance performance showed even greater statistical significance than the final value (SMD = 0.31, 95% CI: 0.15 to 0.46). In the subgroup analysis based on physiological indices, muscle glycogen showed a statistically significant improvement in the protein group (standardized mean difference [SMD] = 0.74, 95% confidence interval [CI]: 0.02 to 0.32). Furthermore, subgroup analyses based on protein supplementation strategies revealed that co-ingestion of protein and carbohydrates (CHO) demonstrated statistically significant improvements in endurance performance (SMD = 0.36, 95% CI: 0.11 to 0.61), whereas high protein intake alone did not.

**Conclusion:**

Protein intake appears to provide modest benefits to athletes in improving their performance, particularly by enhancing endurance. Subgroup analysis suggests that protein intake improves muscle glycogen levels and that the co-ingestion of protein with CHO is more effective for endurance athletes than high protein intake alone.

**Systematic review registration:**

https://www.crd.york.ac.uk/prospero/, Identifier CRD42024508021.

## 1 Introduction

With the rapid development of technology and society, diet and supplements have garnered significant attention from scientists and scholars, particularly in the field of sports. Professional athletes often manage their daily dietary intake under the guidance of dietitians to prepare for upcoming competitions. Protein, as a crucial macronutrient, plays an essential role in human nutrition and warrants further investigation. According to the *NSCA’s Guide to Sport and Exercise Nutrition*, protein can be metabolized for energy, and adequate protein ingestion is especially important for athletes participating in energy-demanding aerobic endurance sports (1).

However, protein is not the body’s preferred fuel source and is metabolized more slowly for energy than carbohydrates CHO. In contrast, CHO is the preferred energy source due to its rapid metabolism, and there is a well-established consensus in sports nutrition that CHO, rather than protein, should constitute the majority of energy intake during prolonged physical activities. Some prestigious authors have supported this viewpoint. Jager et al. concluded that dietary protein is not an ideal energy source and does not enhance endurance performance when adequate CHOs are consumed (2).

According to *ACSM’s Nutrition for Exercise Science*, CHOs have a protein-sparing effect, meaning that protein’s unique roles, such as stimulating muscle protein synthesis (MPS) or reducing muscle protein breakdown, are only activated when the body’s energy needs are met through sufficient CHO intake. Therefore, protein consumption adequacy can only be viewed in the context of whether sufficient total energy has been consumed (3). Phillips and Van Loon concluded that post-exercise protein consumption may enhance adaptation by aiding in glycogen restoration, but this effect seems to occur primarily when carbohydrate intake is insufficient (4).

Proteins are organic compounds composed of a genetically determined sequence of amino acids that serve as protein building blocks. Protein is the diet’s primary source of these essential amino acids (EAAs). Without dietary sources of EAAs, the body must metabolize its protein stores (e.g., muscle) to provide EAAs to meet essential protein needs (1), and intense exercise will increase protein needs (1, 5). People in a general fitness program can generally meet protein needs by ingesting 0.8 to 1.0 g/kg daily. However, competitive athletes, or those who engage in intense training, require more protein than this to adequately respond to the stimulus that training provides (1). The World Health Organization (WHO) suggests an intake of 0.83 g protein/kg weight/day of good-quality protein for all healthy adults of both genders and ages. Typical recommendations for endurance athletes are 1.2 to 1.4 g protein/kg weight/day and for strength-trained athletes from 1.6 to 1.7 g protein/kg weight/day (2, 3, 6).

Although athletes may need more protein to supplement energy, the effect of protein intake on athletic performance is still debatable. The measurement method for athletic performance in cycling and running after a protein intervention is diverse and comprehensive. The completed time, the time to exhaustion (TTE), and the peak power acquired after the time trial (TT) can well reflect running or cycling athletes’ endurance ability and observe their body condition. The consensus is that TTE and TT are well-established endurance performance tests commonly used to examine the influence of experimental interventions on endurance (7, 8). Many studies used these testing methods to explore the relationship between protein ingestion and endurance performance. However, some authors agree that a high-protein diet or supplement may hinder endurance performance in time trials, particularly in activities such as cycling or running (2, 9–12). Additionally, some studies suggest that protein consumption during exercise may not provide immediate ergogenic benefits, especially when carbohydrate intake is limited (13, 14).

Increased protein intake does not necessarily lead to greater power or muscle gain; more data is needed to demonstrate its effectiveness. Athletes who consume excessive protein may experience the opposite results. On the one hand, Rosenbloom et al. determined that increasing protein intake may decrease carbohydrate intake, causing athletes to feel exhausted and perform poorly during training (15). On the other hand, several researchers reported that increased protein intake (higher than or equal to 1.5 g/kg daily) had no additional benefits or drawbacks for athletes’ athletic performance, such as endurance or maximum strength (16–20). Knuiman et al. also mentioned that the evidence of the role of protein on endurance training adaptations and performance is scarce, and there is still no direct evidence that individuals performing endurance training benefit from additional protein (21).

The efficacy of protein plus carbohydrate co-ingestion is also unclear. Some authors concluded that protein plus carbohydrate could not improve athletic performance in athletes compared with carbohydrate ingestion alone (19, 22–25), and protein plus carbohydrate co-ingestion could not enhance muscle glycogen synthesis (26). McCartney et al. discovered that the impact of protein, CHOs, and water on real-world endurance performance in cycling and running was likely negligible, indicating no practical benefits or harms. They recommended prioritizing the consumption of CHOs over protein. The participants included in their study were healthy people (27). Jager et al. stated that protein co-ingestion with additional dietary ingredients may have a favorable impact on muscle strength (13). As a result, it is necessary to collect more data from published studies to investigate the relationship between protein and endurance performance.

On the contrary, some studies have proved the efficacy of protein plus carbohydrate co-ingestion on athletic performance in athletes (28–30), but the source of this benefit is unknown. CHOs are still the primary supplement choice for athletes because they are the main and preferred fuel. Therefore, the performance enhancement resulting from co-ingesting protein with CHOs may be due to the additional energy from either protein or CHOs rather than an isolated effect of the protein itself. It is recommended that a ratio of 3–4:1 of CHO: Protein is optimal for both health and performance in athletic populations (27).

Factors such as blood glucose, heart rate, blood lactate, and muscle glycogen influence athletes’ performance. The extracted data have shown the relationship between protein intake and these factors. A large number of studies have proven the benefits of protein for endurance performance. After the experiment, some scholars mentioned that high protein intake could improve athletic performance and decrease the feeling of fatigue during and/or after exercise, especially in endurance performance, and suggested athletes increase the daily ingestion of protein to 1.5 g/kg a day compared with common people (31–37). Co-ingestion of protein and CHOs could change the perception of exertion by reducing central fatigue, increasing protein oxidation, potentially sparing endogenous CHOs, and enhancing both aerobic and anaerobic endurance under varying Vo_2max_ loads (28–30, 38, 39). A Bayesian meta-analysis (40) found that plant-based protein could improve athletic ability, including muscle strength, endurance performance, and muscle protein synthesis rate in healthy people, but plant-based protein appears to be less effective than other types of proteins, such as beef, whey, or milk protein. Finally, a review (41) summarized the efficacy of dietary protein on endurance and found that periodized protein ingestion has been shown to augment the remodeling of muscle and whole-body proteins with endurance training. Changes in muscle protein synthesis primarily determine protein remodeling, which plays a crucial role in the acute recovery process after exercise and ultimately contributes to the adaptations associated with endurance training, such as increased muscle power and aerobic capacity.

Protein ingestion appears to link with muscle glycogen, an indirect factor that affects athletic performance. In their study, Williams et al. found that ingesting recovery beverages provided following exercises that greatly deplete muscle glycogen stores resulted in an increase in glycogen storage, and the rate of glycogen storage during the CHO co-ingested with PRO treatment was 128% greater than that of the sports beverage treatment. The CHO-PRO beverage contained 53 g of CHOs and 14 g of protein in a serving (355 mL). However, this rise may be attributed to an increase in carbohydrate intake (0.8 g/kg), and further evidence is needed to support the efficacy of protein intake (39).

Rustad et al. found that consuming a protein-plus-carbohydrate beverage immediately after intense exercise accelerated recovery, leading to improved performance 18 h later. The ingestion of CHOs post-exercise was also more beneficial than fasting, possibly due to increased muscle glycogen stores, reduced protein degradation, or a combination of both. In this study, the participants consumed 0.8 g of CHO·kg^−1^·h^−1^ and 0.4 g of whey protein·kg^−1^·h^−1^ (42). Muscle glycogen storage proved to be a key factor in maintaining athletic performance after high-intensity exercise.

Several studies have demonstrated the effectiveness of protein intake in improving muscle strength. A meta-analysis (43) investigated the effect of protein supplements on resistance training-induced gains in muscle mass and strength in healthy adults. They found protein supplements enhanced change in muscle strength and fat-free mass in trained people, but this increase in 1RM and fat-free mass was largely induced by resistance exercise training, and protein intake augmented its efficacy. A network meta-analysis by Lam et al. concluded that whey protein supplements would assist athletes in strength at a longer period of consumption with physical activities (44). In the same way, a review exploring the effect of protein on athletic performance concluded that beef protein and whey protein combined with exercise training both could improve lower limb muscle strength, and this increase seems to be attributed to the better stimulation of MPS by protein (45). Bagheri et al. concluded that an intake of 1.6 g/kg a day of protein combined with resistance training appeared sufficient to maximize gains in lean mass, muscle strength, performance, and aerobic capacity, indicating this daily protein amount is effective and safely tolerated in young, healthy adults. Participants consumed 40 g of an isolated whey protein beverage containing 110 calories upon cessation of every training session (46). High-protein dairy milk combined with resistance training could effectively increase total energy intake and augment lean mass and muscle performance in young resistance-trained males already ingesting 1.5 g/kg of protein. Participants ingested 250 mL of 156 kcal high-protein dairy milk containing 30 g of a mixture of whey (6 g) and casein (24 g) with 10 g carbohydrate (47), and Fritz et al. concluded that vegan protein supplements with probiotics improved body weight and skeletal muscle mass in 19 players. The improved body weight and skeletal muscle mass were probably attributed to the change in the gut microbiota composition and better protein absorption. The composition of Biotech vegan protein contains pea protein, rice protein, and soy lecithin (48). Therefore, it is meaningful to combine studies with different results and data through meta-analysis to provide robust conclusions in the field of protein supplements.

When athletes, especially runners or cyclists, eat protein after a TTE or TT simulated test, their endurance may improve. This could provide benefits such as allowing them to perform longer in a race. This improvement may be linked to protein oxidation, extra energy, or less central fatigue. For instance, Highton et al. concluded that the ability to sustain high-intensity running is particularly relevant for sprint athletes, and CHOs with protein beverages could provide a meaningful (small to moderate) advantage over CHOs at the phase of a multiple-sprint sport in which fatigue-related performance deterioration is most likely to have an impact. Therefore, they likely attributed the enhanced performance to either altered central fatigue or increased protein oxidation (28). Another study has provided the same conclusion. Saunders et al. summarized that cyclists who ate protein-carbohydrate gels could decrease the incidence rate of muscle injury, prolong the time to exhaustion, and increase protein oxidation. Therefore, protein’s ergogenic effects may come from raising the upper limit of exogenous caloric uptake or oxidation above what can be done with treatments that only use CHOs (38).

Currently, there is a lack of comprehensive evidence regarding the impact of protein on athletes’ athletic performance. Few studies have investigated the efficacy of protein ingestion on athlete populations, particularly in endurance athletes, and have reached a clear conclusion. Jager et al. described that very few studies have investigated the effects of prolonged periods (1 week or more) of dietary protein manipulation on endurance performance (13). Therefore, it is necessary to synthesize all available evidence and arrive at a robust conclusion.

This meta-analysis aims to examine athletes’ physiological responses to protein intake from various sources, including food bars, whey protein, plant protein, and other supplements. By synthesizing current evidence, this study seeks to provide clearer insights into the effectiveness of protein on athletic performance. We hypothesize that protein intake can improve athletic performance through muscle strength and endurance performance in athletes. The results of this study may have practical implications for athletes and their advisors in planning protein supplements to enhance competitive ability.

## 2 Method

This study was registered in PROSPERO (CRD42024508021) and reported in accordance with PRISMA guidelines. We performed a meta-analysis using Review Manager 5.3, Stata 12, Get Data Graph Digitizer 2.26, and SPSS.

### 2.1 Search strategy

In January 2024, 1,246 studies were extracted by two authors (S.Z. & Y.X.) from 5 databases, including Web of Science, Ovid, Scopus, Pubmed, and Ebsco, into the software Endnote X9 to be screened, and no more studies were from other resources. The keywords and subject heading were determined through two reviewers’ discussions, and these words were collected from the Pubmed Mesh database. The confirmed words were as follows: “High protein, diet OR Plant protein, dietary OR Vegetable protein OR Whey protein OR Egg protein OR White protein OR Amino acid OR athletic performance OR Sports performance OR Endurance performance OR Muscle strength.” Each previously mentioned database utilized these terms.

### 2.2 Inclusion and exclusion criteria

We excluded non-human studies, including those on animals, plants, and so on. The studies without a control group or protein group were not considered. Studies that lacked available data to be extracted and non-original studies, including letters, reviews, or editorials, were excluded during the selection process. The studies that used other languages instead of English were excluded.

We included the randomized controlled trial, which included male and female athletes. The included studies must include both a control group and a protein group, with the control group demonstrating a significant difference in protein intake compared to the protein group. Studies must provide quantitative measurements of athletic performance or physiological indices, such as aerobic performance, anaerobic performance, heart rate, blood glucose, and so on.

### 2.3 Selection process

**Figure 1:**
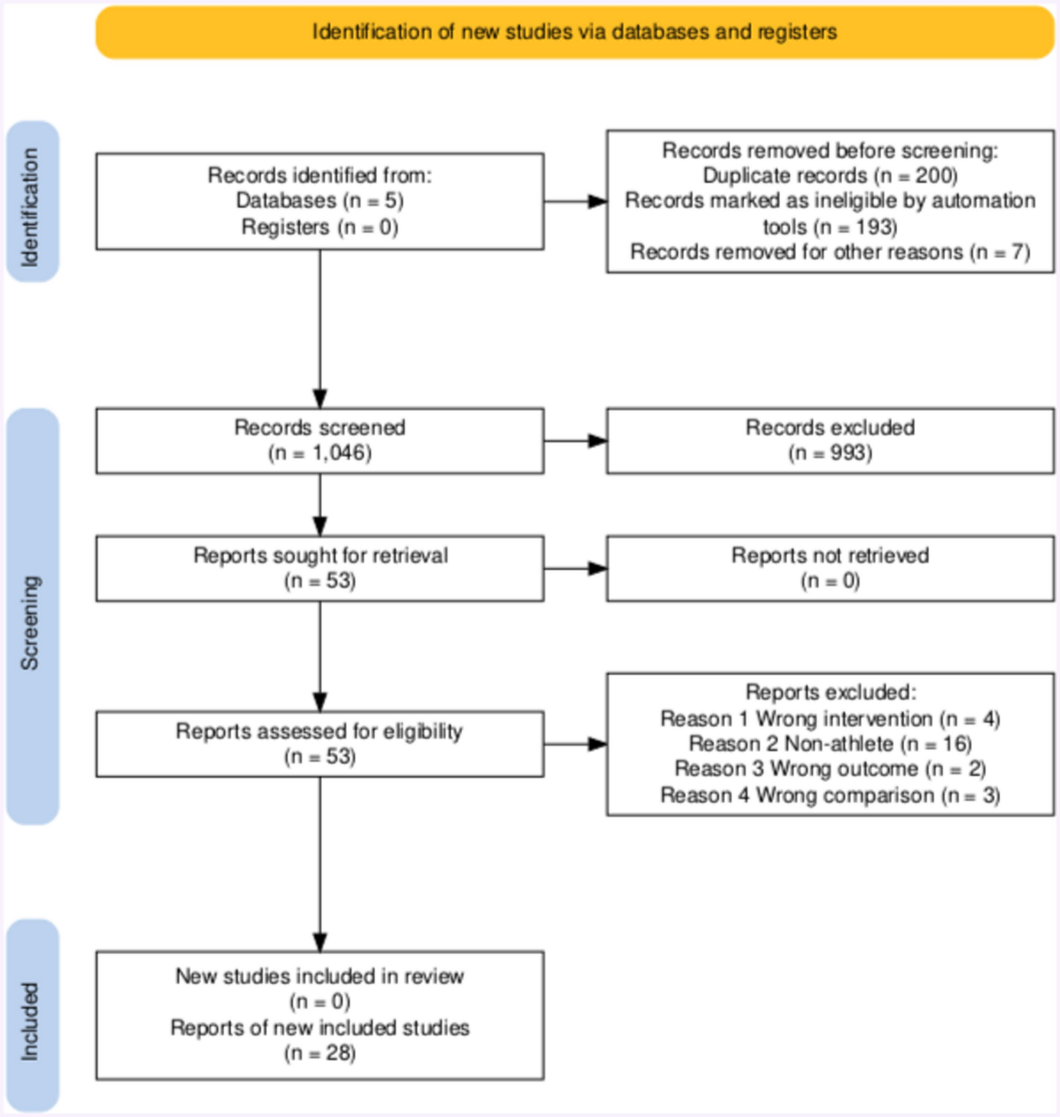
Prisma flow chart for the identification of the included studies.

Figure 1 shows the selection process and information sources. The automatic tool recommended by PRISMA was used to make the flow chart (49). The literature selection started in February 2024. Two authors, S.Z. and Y.X., independently searched and evaluated the literature using the previously confirmed inclusion and exclusion criteria to exclude duplicates. The Endnote auto tool initially eliminated duplicate literature, followed by screening by two authors. In the first step, 1,046 articles were screened based on their title and abstract, marking 993 articles as irrelevant. We advanced 53 articles to the next step for further full-text assessment, while 25 articles were excluded due to data ambiguity and participant irrelevance. During the full-text screening process, two articles on plant-based protein with more than 50 participants were discovered, but they were not chosen for the final data extraction and meta-analysis due to the absence of a comparable group. The meta-analysis ultimately included 28 articles.

### 2.4 Study risk of bias assessment

The risks of bias for all included studies were independently assessed using the guidelines and criteria outlined in the Cochrane Handbook for Systematic Reviews of Interventions. Two authors (S.Z. & Y.X.) assessed the included literature through the Cochrane risk of bias (ROB) assessment tool. Seven areas of bias risk were assessed: (1) random sequence generation; (2) allocation concealment; (3) blinding of participants and personnel; (4) blinding of outcome assessment; (5) incomplete outcome data; (6) selective reporting; and (7) other bias. The risk of bias was classified into low, high, and unclear. Two reviewers performed the Kappa consistency test using the software SPSS after the quality assessment. If the Kappa results were poor, we asked the third or fourth reviewers (S.D. & J.L.) to reassess the quality of all included studies and discuss how to correctly adjust the quality assessment results until we got a good Kappa value.

After the data extraction process and meta-analysis, the risk of bias was assessed using the funnel plot and the *p* value from Egger’s and Begg’s tests. Funnel plots were generated in Stata 12.0, displaying the symmetry of the included data, with circle dots distributed evenly on both sides of the plot. A *p*-value less than 0.05 in both Egger’s and Begg’s tests indicated no significant risk of bias. Any discrepancies between reviewers at any stage of the process were resolved through discussion and consensus. The final data were presented in the results section.

### 2.5 Certainty in evidence

GRADEprofiler software was used to assess each main result. The quality of protein evidence was assessed using the GRADE approach, which provided a clear method to rate the quality across studies by evaluating the risk of bias, inconsistency of results, indirectness, and imprecision of effect estimates. The GRADE approach classifies the quality of evidence as high, moderate, low, or very low.

### 2.6 Data extraction and analysis

Two authors collected data using Endnote X9. The authors appropriately extracted the eligible data into the Excel template. The original data types were mean plus standardized deviation, mean plus standardized error, or mean plus confidential interval. Data were presented as mean plus standard deviation (M ± SD). Review Manager was used to convert data not initially in M ± SD format.

The information we extracted was as follows: country, author, publication years, participants’ age, total number, gender, classification of athletes, and types of dietary interventions (control and experimental group), and the available outcome data included the time to exhaustion, peak power, deadlift, squat, blood glucose, Vo_2max_, heart rate, blood lactate, bench press, maximum voluntary contraction (MVC), Wingate test, counter-movement jump (CMJ), running average speed, and maximum speed. Physiological indices include blood glucose, muscle glycogen, heart rate, and blood lactate.

All of the data collected were continuous variables. The effect sizes calculated in the meta-analysis were standardized mean difference (SMD) or mean difference (MD). When the article did not provide accurate data, the Get Data Graph Digitizer software extracted data from the graph, including the desired outcomes. We divided all original data from the 28 articles into two parts. One was the mean change difference and corresponding standard deviation (ΔSD) of the outcomes of interest from text, tables, and graphs to compare the changes through interventions between the protein and control group. The other was the final value after the intervention to compare the difference between the two groups. The data were mean plus standardized deviation (M ± SD). When the ΔSD was not reported in the study, we estimated the ΔSD by calculating the correlation coefficient (corr) according to the formula provided by the Cochrane Handbook for meta-analysis of intervention:


$$\mathrm{Corr}=\left({\mathrm{SDpre}}^{2}+{\mathrm{SDpost}}^{2}-{\mathrm{SDchange}}^{2}\right)/\left(2\times \mathrm{SDpre}\times \mathrm{SDpost}\right)$$

The ΔSD was then calculated using the formula:


$$\Delta SD=\surd \left({\mathrm{SDpre}}^{2}+{\mathrm{SDpost}}^{2}-2\times \mathrm{corr}\times \mathrm{SDpre}\times \mathrm{SDpost}\right)$$

All of the data were exported into an Excel template to make the literature characteristic chart, and we also used it to make a forest plot and did the test for heterogeneity to calculate the I^2^value to represent. All forest plots chose the fixed-effect model because the calculated heterogeneity I^2^ < 50%. In the meta-analysis, some articles used three groups, one control group and two experimental groups, to carry out their research. To address this, we compared the control and two experimental groups in the forest plot, respectively, which means we used the control group’s data twice.

### 2.7 Subgroup analysis

Despite the relatively low heterogeneity, subgroup analysis was conducted to fully understand the impact of protein intake on athletic performance. Subgroup analysis was divided into several parts: (1) subgroup analysis based on the types of athletic performance; (2) subgroup analysis based on protein supplementation strategy; (3) subgroup analysis based on each athletic performance test and blood parameters; and (4) subgroup analysis based on energy matching and the amount of protein ingestion (1.5 g/kg daily or 1.5 g/kg daily).

### 2.8 Sensitive analysis

Stata 12.0 conducted a sensitive analysis to evaluate the credibility and robustness of all included data. The sensitive analysis used the leave-one-out method to assess whether the study’s included data was robust.

## 3 Results

### 3.1 Study characteristics

All studies included in the meta-analysis were randomized controlled trials (RCTs). This meta-analysis included the randomized crossover design (RCD), a type of RCT. 10 studies were RCD. Tables 1, 2 provide the details and characteristics of the 28 studies. This meta-analysis analyzed data from 373 participants. A total of 14 studies included cyclists as participants, while one study (18) did not specify the type of athletes involved. Participants in 2 studies were soccer players (28, 31), and 2 studies’ participants were triathletes (23, 33). Other participants were rugby players, sailing players, and climbing players (28, 32, 36). The majority of participants in the included studies were male, and three studies did not collect information on participants’ gender. The publication years spanned from 2001 to 2023, and the participants were from 13 countries on the following continents: Europe, North America, and South America.

**Table 1:** The characteristics of the included studies.

Code	Region	Author	Year	Study design	Sample	Sport Subjects	Gender (Male/Female)	Age (Mean ± SD)	Amount of protein
1	UK	Highton	2012	RCD	9	Soccer & rugby players	9/0	23.4 ± 1.2	0.5 g/kg daily
2	Australia	Hall	2012	RCD	10	Cyclists	10/0	29.7 ± 5.01	0.54 g/kg daily
3	Malaysia	Ghosh	2010	RCD	8	Cyclists	8/0	21.5 ± 1.1	0.3 g/kg daily
4	Switzerland	Furber	2021	RCT	16	Endurance runners	16/0	26 + 4.5	4.6 g/kg daily
5	USA	Grubic	2019	RCD	12	Resistance-trained athletes	12/0	22 ± 1.8	0.73 g/kg daily
6	USA	Saunders	2007	RCT	13	Cyclists	8/5	24.2 ± 6.8	0.038 g/kg
7	UK	Röhling	2021	RCT	23	Endurance athletes	16/7	Not clear	0.75 g/kg daily
8	USA	Schroer	2014	RCT	8	Cyclists	4/4	22.3 ± 5.6	1.93 g/kg daily
9	New Zealand	Rowlands	2011	RCD	12	Cyclists	0/12	30 ± 7	2.8 g/kg daily
10	UK	Witard	2011	RCD	8	Cyclists	8/0	27 ± 8	3 g/kg daily
11	Greece	Kritikos	2021	RCD	10	Soccer players	10/0	21 ± 1.5	1.5 g/kg daily
12	France	Portier, H.	2008	RCT	12	Sailing players	12/0	36.15 + 8.85	3.2 g/kg daily
13	USA	Campbell	2018	RCT	17	NA	0/17	21.2 ± 2.1	2.5 g/kg daily
14	Canada	Naclerio	2017	RCT	24	Triathletes	24/0	46.17 + 7.98	1.6 g/kg daily
15	UK	Furber	2022	RCT	16	Endurance runners	Not clear	26.6 + 4.33	4.6 g/kg daily
16	Not clear	Laskowski	2003	RCT	12	Judo players	Not clear	16.25 + 2.33	0.50 g/kg daily
17	UK	Mettler	2010	RCT	20	Not clear	20/0	25.25 + 5.11	2.31 g/kg daily
18	France	Bourrilhon	2010	RCD	10	Climbing Players	10/0	30 ± 2.85	4.1 g/kg daily
19	Brazil	Finger	2018	RCT	13	Triathletes	13/0	29.7 ± 7.7	0.3 g/kg daily
20	UK	Naclerio	2019	RCT	25	Endurance athletes	25/0	32.28 + 8.35	0.3 g/kg daily
21	UK	Toone	2010	RCT	12	Cyclists	12/0	23.4 ± 3.2	0.3 g/kg daily
22	New Zealand	Thomson	2011	RCT	10	Cyclists	10/0	33 ± 9	0.75 g/kg daily
23	Spain	Valenzuela	2023	RCT	24	Cyclists	Not clear	19.33 + 1.73	3.5 g/kg daily
24	Denmark	Hansen	2016	RCT	18	Cyclists	18/0	19.5 + 2	0.95 g/kg daily
25	Norway	Sollie	2018	RCD	8	Cyclists	8/0	22.9 ± 3.39	0.76 g/kg daily
26	New Zealand	Macdermid	2006	RCT	7	Cyclists	7/0	33.6 ± 5.0	3.3 g/kg daily
27	USA	Williams	2003	RCD	8	Cyclists	8/0	28.4 ± 4.8	0.5 g/kg daily
28	UK	Jentjens	2001	RCT	8	Cyclists	8/0	27.1 ± 7.35	0.85 g/kg daily

**Table 2:** The characteristics of diet intervention and athletic performance measure method.

Code	Author	Diet intervention			Athletic performance measure method
		Control group	Experiment group		
1	Highton	CHO	CHO + PRO		Modified Loughborough intermittent shuttle test
2	Hall	CHO	CHO + PRO		VO2max test; cycling time trial; Blood sampling
3	Ghosh	PLA	CHO	CHO + PRO	VO2max test; Time to exhaustion test; Blood sampling
4	Furber	CHO	PRO		Running distance and speed; Cycling time trial; and Blood sampling
5	Grubic	CHO	CHO + PRO		Maximum voluntary contraction (MVC); Resistance exercise; Sprint performance and Blood sampling
6	Saunders	CHO	CHO + PRO		Physical fitness assessment Experimental ride with blinded treatment
7	Röhling	PLA	PRO		Running completed time and speed; VO2max test; and Blood sampling
8	Schroer	PLA	ALA	PRO	Cycling time trial; VO2max test; and Blood sampling
9	Rowlands	CHO	PRO		Sprint performance, strength performance, and Blood sampling
10	Witard	Normal diet	PRO		VO2max test; Cycling time trial; and Blood sampling
11	Kritikos	PLA	Whey Protein	Soy protein	Speed-endurance training, MVC, and Blood sampling
12	Portier	Normal diet	PRO		Physical performance test (Jump test and Handgrip Strength)
13	Campbell	Low protein diet	PRO		Maximal strength Resistance and high-intensity interval training and blood sampling
14	Naclerio	CHO	Beef protein	Whey protein	VO2max test
15	Furber	CHO	PRO		Time trial to exhaustion performance
16	Laskowski	Normal diet	PRO		VO2max test; Wingate test (peak power)
17	Mettler	Normal diet	PRO		MVC; jump test; Wingate test (peak power); Blood sampling
18	Bourrilhon	High carbohydrate diet	PRO		MVC
19	Finger	PLA	CHO	CHO + PRO	Cycling test; Running test
20	Naclerio	CHO	CHO + PRO		VO2max test; maximal aerobic speed
21	Toone	CHO	CHO + PRO		Cycling time trial; blood sampling
22	Thomson	Normal diet	PRO		Maximum aerobic power; repeat-sprint performance test
23	Valenzuela	CHO + PLA	Pre-sleep protein	Afternoon protein	Counter-movement jump (CMJ); cycling time trial
24	Hansen	CHO	CHO + PRO		10 s peak power test; 5 min all-out performance test
25	Sollie	CHO	CHO + PRO		Time to exhaustion test; Sprint power; Blood sampling
26	Macdermid	CHO	PRO		Time Trial Performance; Blood sampling
27	Williams	CHO	CHO + PRO		Time to exhaustion test; Blood sampling
28	Jentjens	CHO	CHO + PRO		Graded exercise test to exhaustion; Blood sampling

Twelve studies’ intervention methods were carbohydrate (CHO) plus protein (PRO) co-ingestion. Six studies had three groups: one control group and two experimental groups. Five studies used a placebo (PLA) group as the control group. The difference in protein content was the main distinction between the control and experimental groups.

### 3.2 Quality assessment

**Figure 2:**
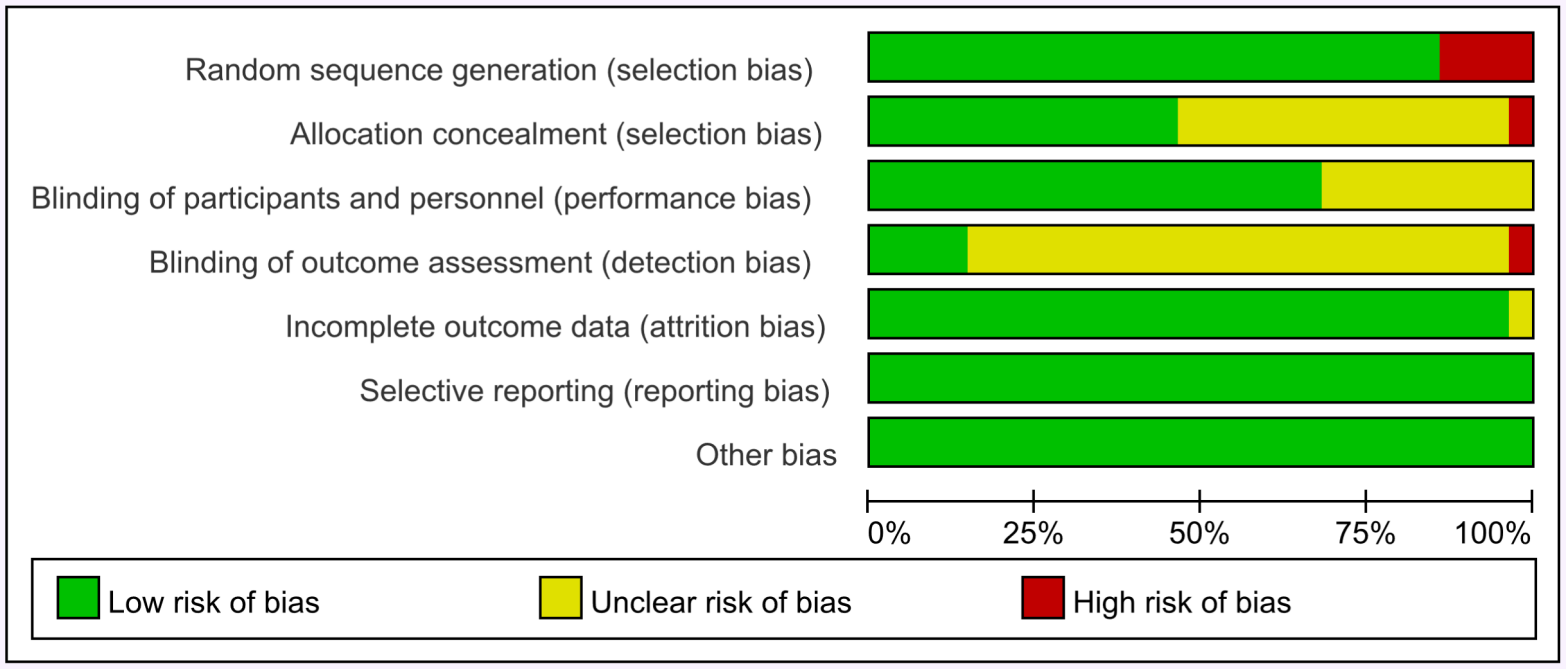
Risk of bias summary.

**Figure 3:**
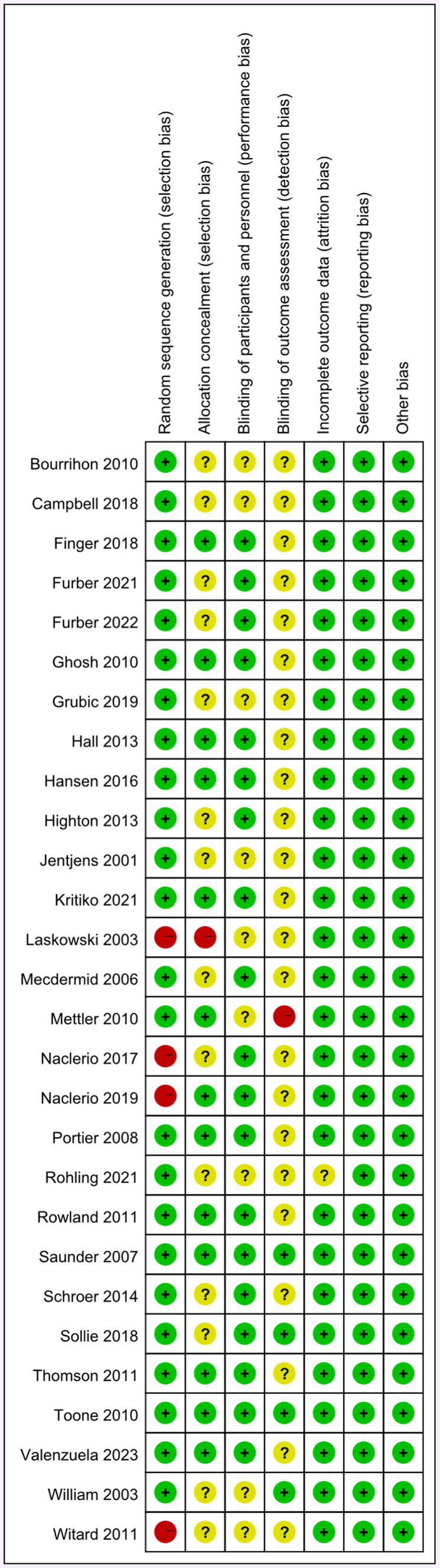
Risk of bias graph.

The details of the quality assessment can be seen in Figures 2, 3. The risk of bias in the included studies was assessed using the ROB scale, and the results were presented using the software Reviewer Manager 5.3. Some studies’ randomized methods were insufficient. The use of incomplete random allocation methods, such as block randomization, resulted in these studies being classified as high risk (4 articles, accounting for 14%) (24, 33, 34, 50). Some studies did not provide enough information on allocation concealment (57%), and one study did not conceal the allocation process to participants (3.5%) (34). Some studies failed to provide comprehensive details about participant and personnel blinding (32%), while 82% of the included studies lacked sufficient information on outcome data blinding, resulting in their classification as unclear risk. One study (35) was marked as high risk (3.5%) due to its failure to conceal the outcome data from participants and researchers during the test. Only one study (16) incurred an unclear risk of incomplete data due to participant withdrawal during the experiment (3.5%).

After two reviewers (S.Z. & Y.X.) assessed all of the included studies, we did the Kappa consistency test. The result was 0.286 at first, so we invited the third scholar (S.D.) to be the reviewer to reassess all studies. After three reviewers’ discussions, we obtained the final result. The final Kappa value was 0.761, indicating excellent agreement in quality assessment between reviewers. Table 3 illustrates this.

**Table 3:** The Kappa consistency test of quality assessment.

Kappa				
	Value	Asymptotic standard error	Approximate *T*	Approximate significance
Measurement of agreement	0.761	0.063	12.85	0.0001
*N* of valid cases	196			

### 3.3 Quality grade in each main outcome

**Figure 4:**
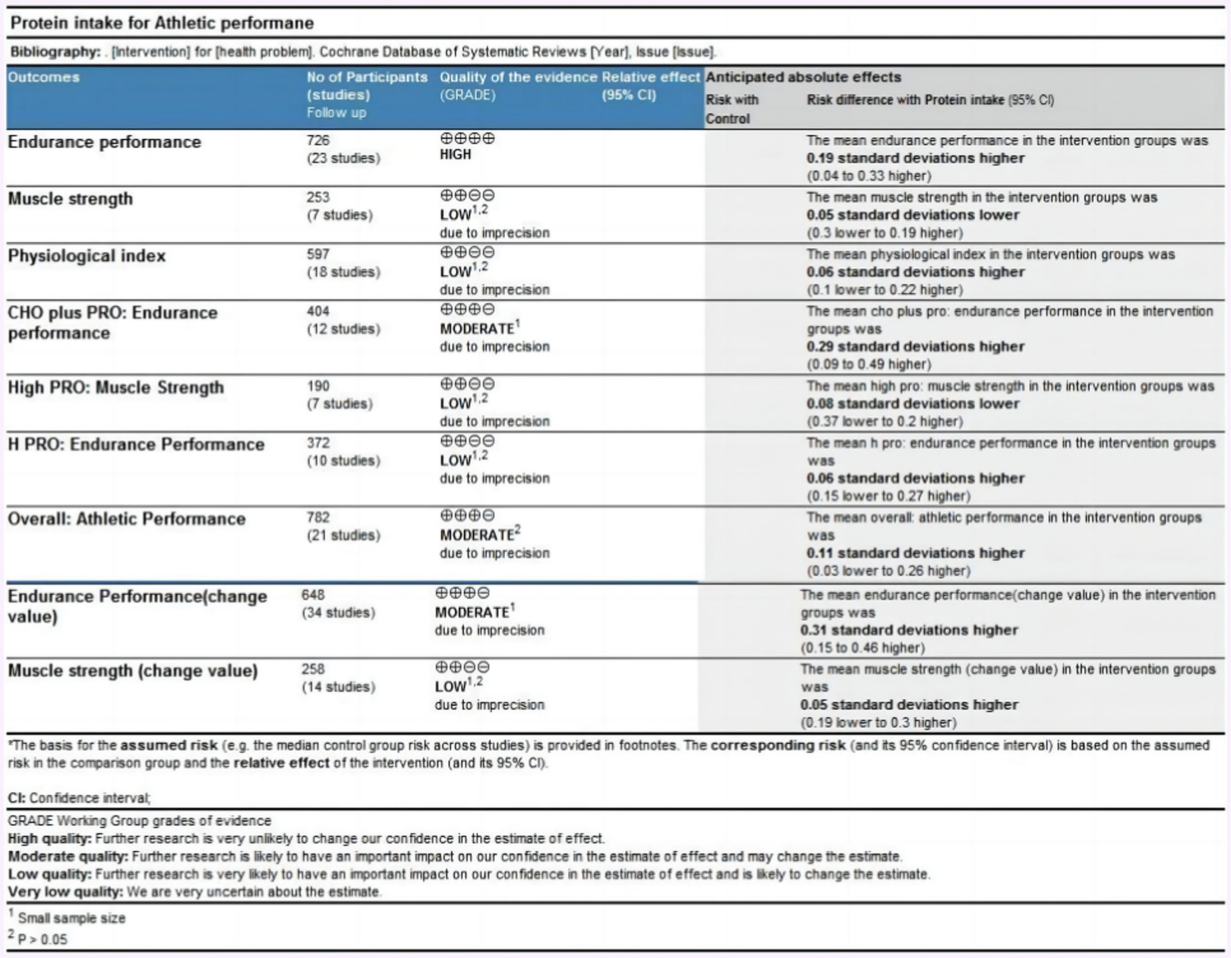
The quality grade of athletic performance.

Data from nine outcomes were assessed (Figure 4). The outcome of endurance performance was rated as high quality. The outcome of athletic performance was rated as moderate quality due to statistical insignificance. The outcomes of muscle strength, physiological index, and muscle strength presented by change value were rated as low quality due to the small sample size and statistical insignificance. The outcomes of muscle strength and endurance performance intervened by high protein ingestion were rated as low quality due to the small sample size and statistical insignificance. The outcome of endurance performance intervened by protein plus carbohydrate ingestion was rated moderate due to the small sample size. The outcome of endurance performance presented by the change value was rated moderate due to the small sample size.

### 3.4 Meta-analysis

**Figure 5:**
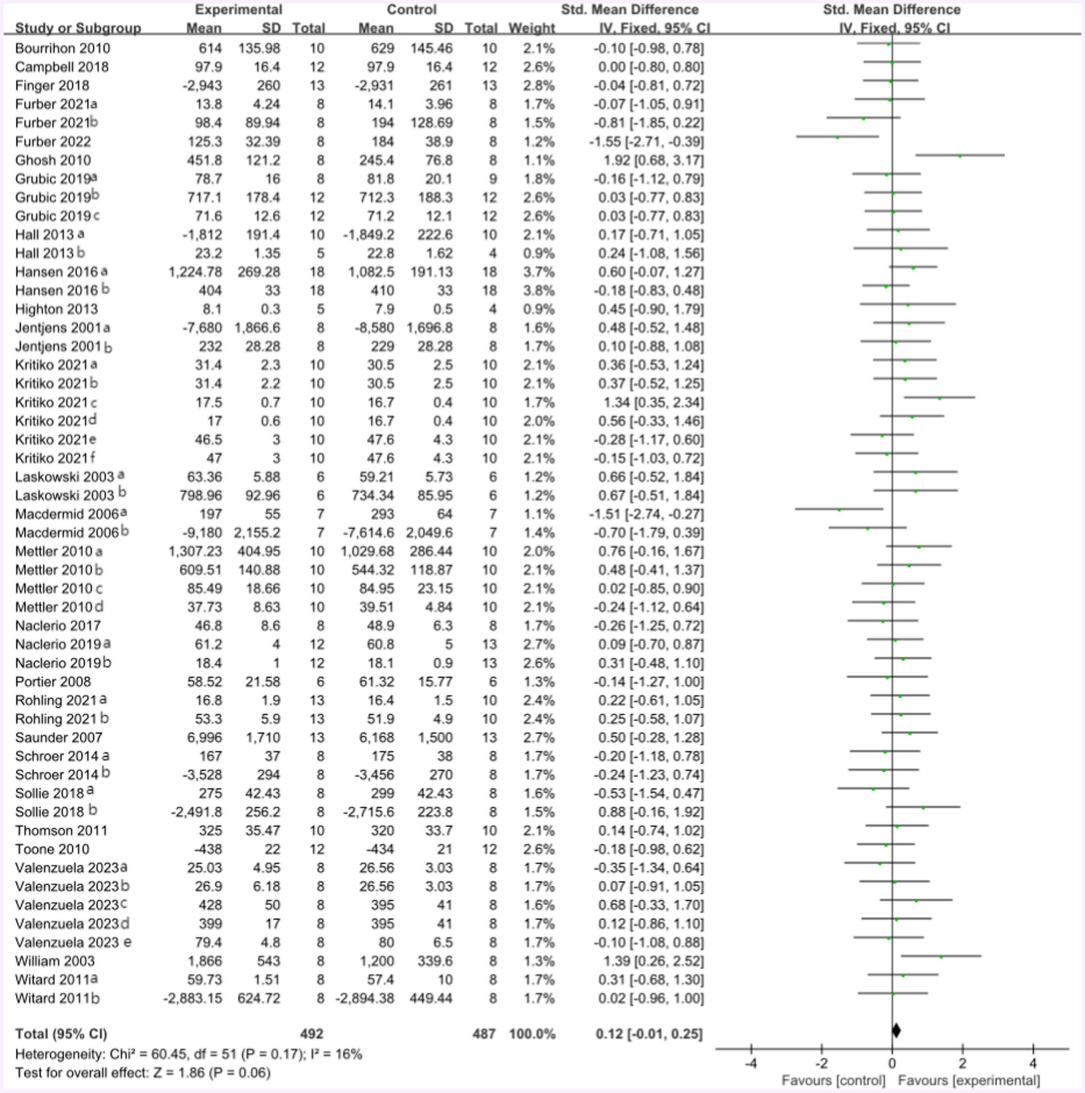
The forest plot of athletic performance (no protein group vs. protein group, and final value).

All included studies compared the effect of the protein group vs. the non-protein group on athletic performance. A total of 27 studies provided 361 participants’ athletic performance data in the forest plot presented in Figure 5 (final value), including the running maximum speed and average speed, peak power in the Wingate test, the time to exhaustion of cycling with different percentages of Vo_2max_, Vo_2max_, cycling completed time, and mean power in time trial, 1RM, MVC, jump test, and CMJ. The mean athletic performance effect size was 0.12 with a 95% confidence interval of −0.01 to 0.25, *p* = 0.06, Z = 1.86, and I^2^ = 16%. The effect of the control and protein groups on athletic performance has not been observed with any statistical significance.

### 3.5 Subgroup analysis

The subgroup analysis was divided into five parts: (1) subgroup analysis based on athletic performance types; (2) subgroup analysis based on protein supplementation strategies; (3) subgroup analysis based on each athletic performance test and blood parameters; (4) subgroup analysis based on energy matching or not between protein group and non-protein group; and (5) subgroup analysis based on the amount of daily protein ingestion in the protein group (1.5 g/kg daily or 1.5 g/kg daily). Parts 1 to 3 aimed to explore the effect of protein intake on different types of athletic performance. Parts 4 and 5 aimed to avoid imprecision in the meta-analysis because of the difference in energy matching or the amount of protein ingestion.

#### 3.5.1 Subgroup analysis based on the amount of daily protein ingestion in protein group (<1.5 g/kg daily or ≥ 1.5 g/kg daily)

Table 4 presents the subgroup analysis based on protein intake. The protein ingestion that was lower than 1.5 g/kg daily showed statistical significance, and the protein ingestion that was higher than or equal to 1.5 g/kg daily showed insignificance. Thirteen of the fifteen studies implemented a carbohydrate plus protein intervention, while all twelve implemented a high protein intervention. This subgroup analysis result aligns with the subgroup analysis based on the different types of protein supplementation strategies. Thus, the difference in the amount of protein ingestion did not affect the result of this meta-analysis or cause deviation.

**Table 4:** Subgroup analysis based on the amount of protein ingestion (<1.5 g/kg daily or ≥ 1.5 g/kg daily).

Subgroup name	Studies number	SMD (95% CI)	*p*	*Z*	I^2^
Protein ingestion (lower than 1.5 g/kg daily)	15	0.24 (0.06 to 0.42)	0.008	2.63	0%
Protein ingestion (higher than or equal to 1.5 g/kg daily)	12	0.00 (−0.17 to 0.18)	0.98	0.02	19%

#### 3.5.2 Subgroup analysis based on the types of athletic performance

Five forest plots (final value & change value) present three mean effect sizes of endurance performance, muscle strength, and physiological indices. Three forest plots included the final value data, while two included the change value data. The summary of the overall effect size can be seen in Table 5 (Final value) and Table 6 (Change value).

**Table 5:** The summary of different athletic performance (final value).

Name	No. of studies	No. of participants	Effect size (SMD)	*p*	*Z*	I^2^
Endurance performance	23	310	0.19 [0.04, 0.33]	0.03	2.42	31%
Physiological index	18	206	0.06 [−0.10, 0.22]	0.48	0.71	0%
Muscle strength	7	105	−0.05 [−0.30, 0.19]	0.68	0.42	0%

**Table 6:** The summary of different athletic performance (change value).

Name	No. of studies	No. of participants	Effect size (SMD)	*p*	*Z*	I^2^
Endurance performance	17	272	0.31 [0.15, 0.46]	0.0001	3.81	0%
Muscle strength	9	105	−0.05 [−0.19, 0.30]	0.67	0.42	0%

**Figure 6:**
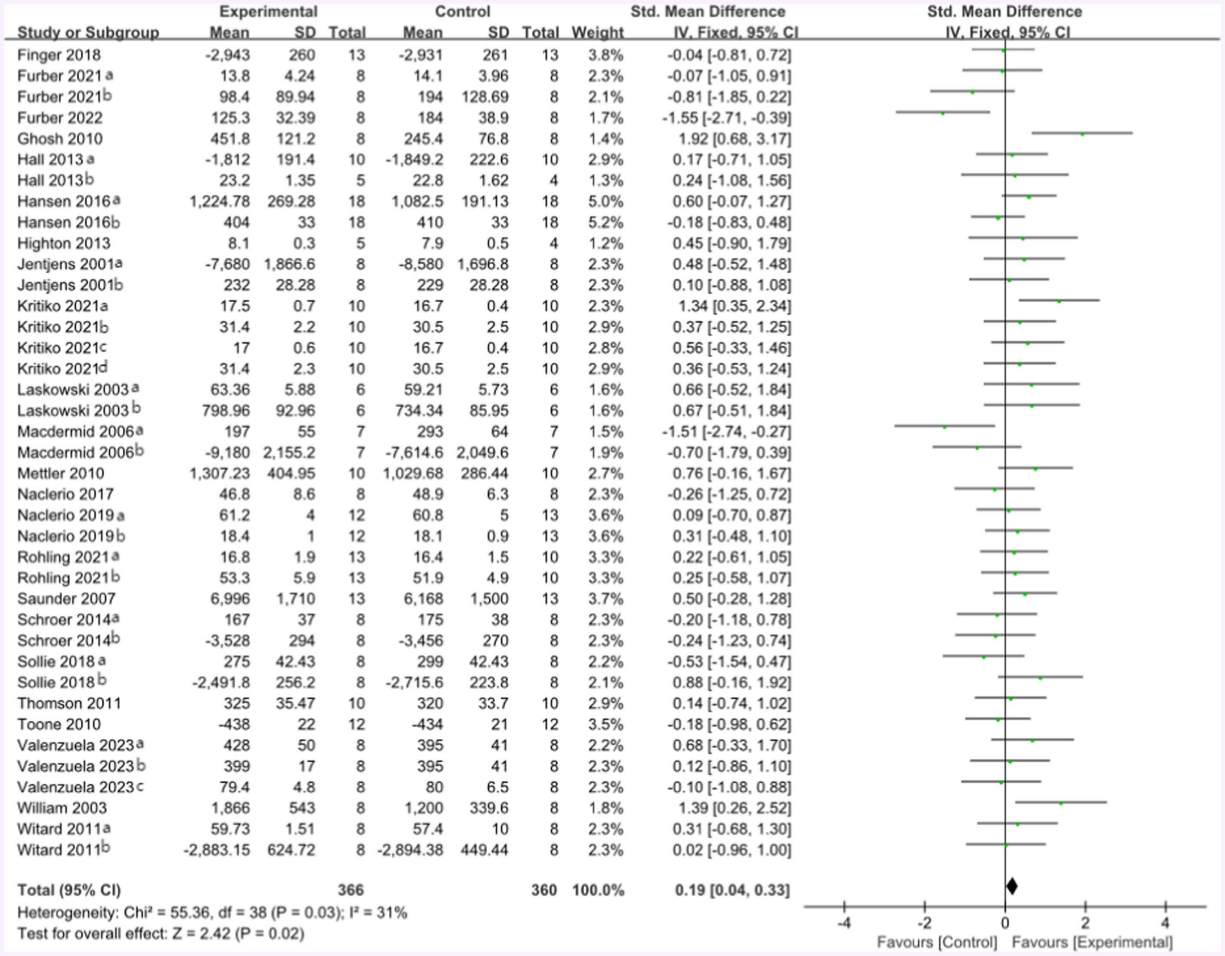
The forest plot of endurance performance (no protein group vs. protein group and final value).

The mean endurance performance effect size was 0.19 with a 95% confidence interval of 0.04 to 0.33, *p* = 0.02, Z = 2.42, and I^2^ = 31%. Compared with the control group, the protein group had a greater gain in endurance performance, which includes aerobic and anaerobic capacity (Figure 6).

**Figure 7:**
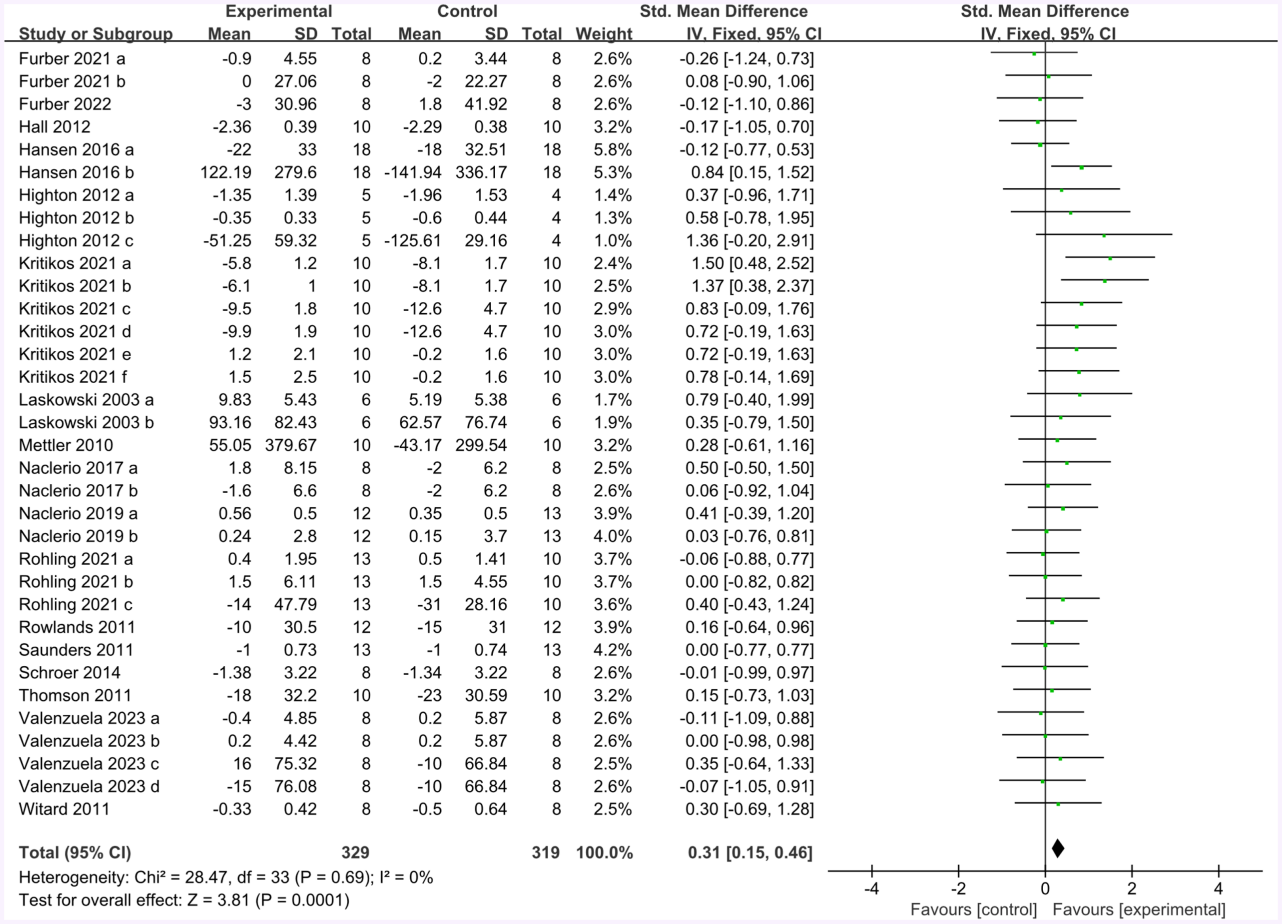
The forest plot of endurance performance (no protein group vs. protein group and change value).

Seventeen studies provided 272 participants’ data about endurance performance in the forest plot presented in Figure 7 (change value). The mean endurance performance effect size was 0.31 with a 95% confidence interval of 0.15 to 0.46, *p* = 0.0001, Z = 3.81, and I^2^ = 0%. Compared with the control group, the protein group had a greater gain in endurance performance, which includes aerobic and anaerobic capacity.

**Figure 8:**
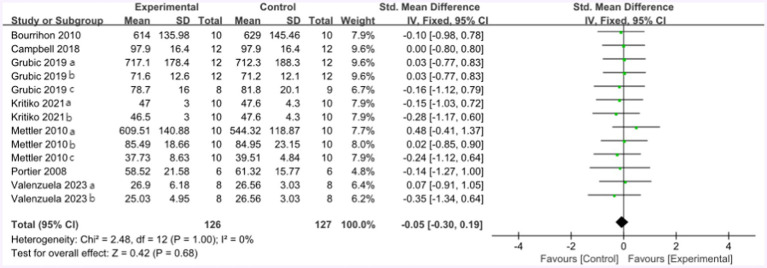
The forest plot of muscle strength (no protein group vs. protein group, final value).

Figure 8 summarized the mean effect size of muscle strength, which included MVC, jump height, 1RM chest press, 1RM bench press, and 1RM squat. This forest plot included seven studies and 105 participants. The mean muscle strength effect size was −0.05 with a 95% confidence interval of −0.3 to 0.19, *p* = 0.68, Z = 0.42, I^2^ = 0%. No difference between the protein and control groups was observed.

**Figure 9:**
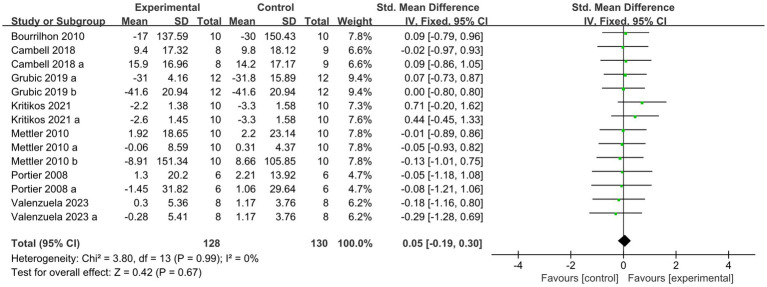
The forest plot of muscle strength (no protein group vs. protein group and change value).

In the forest plot shown in Figure 9 (change value), nine studies provided 105 participants’ muscle strength data. The mean muscle strength effect size was −0.05 with a 95% confidence interval of −0.19 to 0.3, *p* = 0.67, Z = 0.42, I^2^ = 0%. No difference between the protein and control groups was observed.

**Figure 10:**
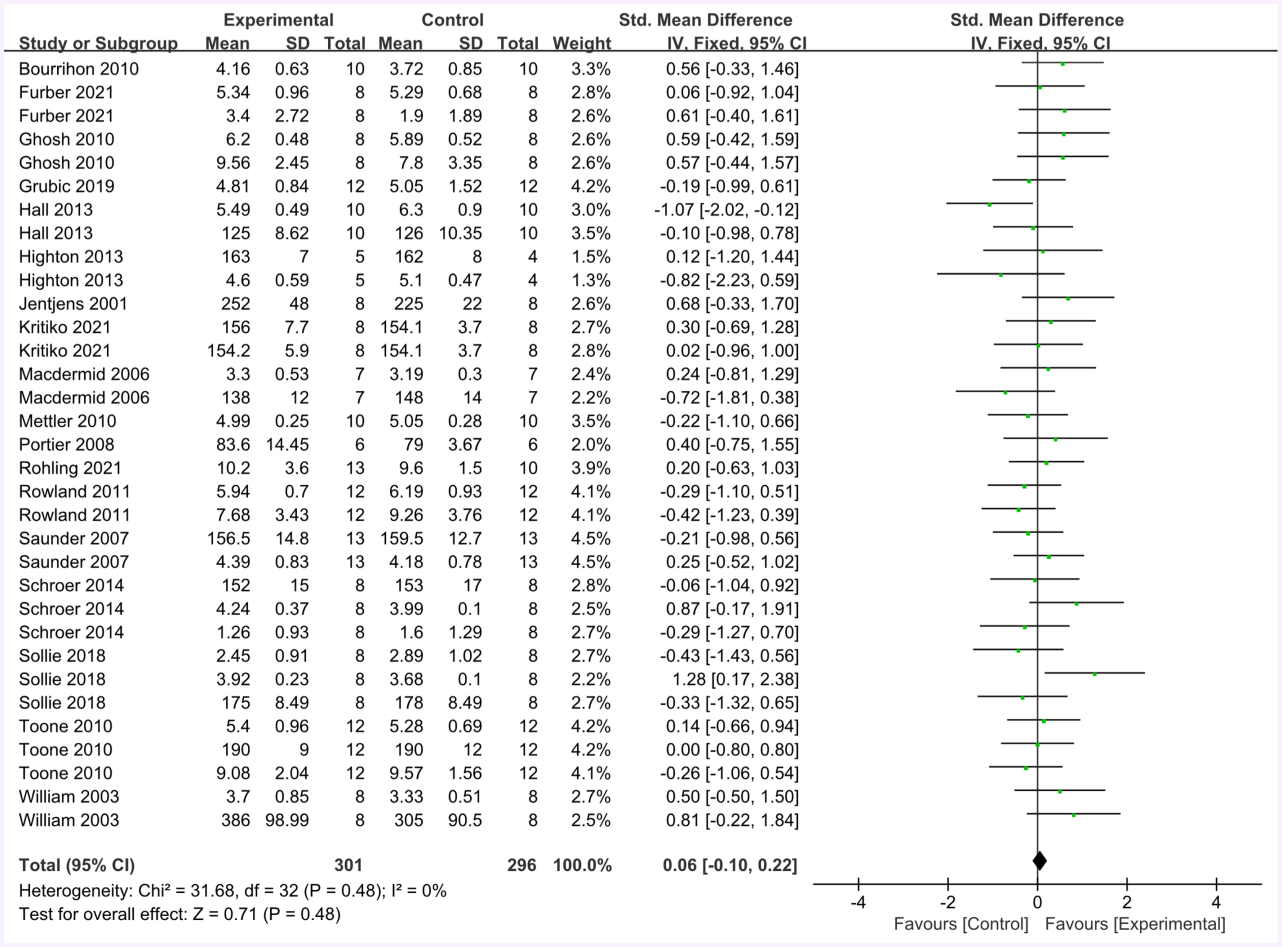
The forest plot of physiological indices (no protein group vs. protein group, final value difference).

Figure 10 summarizes the mean effect size of the physiological indices, which include blood glucose, blood lactate, heart rate, and muscle glycogen. This forest plot included 18 studies and 206 participants. The mean physiological index effect size was 0.06 with a 95% confidence interval of −0.10 to 0.22, *p* = 0.48, Z = 0.71, I^2^ = 0%. No difference between the protein and control groups was observed.

#### 3.5.3 Subgroup analysis based on protein supplementation strategy

Table 7 summarizes the subgroup analysis based on the different protein supplementation plans. To conduct the subgroup analysis, the protein supplementation program was divided into two sections: (1) Protein plus Carbohydrate (PRO+CHO) and (2) High Protein intake.

**Table 7:** The summary of subgroup analysis based on protein supplementation strategy (final value).

Subgroup name	The types of athletic performance	SMD (95%CI)	*p*	*Z*	I^2^
Protein plus carbohydrate	Endurance performance	0.36 [0.11, 0.61]	0.005	2.78	2%
High protein intake	Endurance performance	0.18 [−0.01, 0.37]	0.07	1.82	17%
High protein intake	Muscle strength	−0.08 [−0.37, 0.2]	0.56	0.58	0%

**Figure 11:**
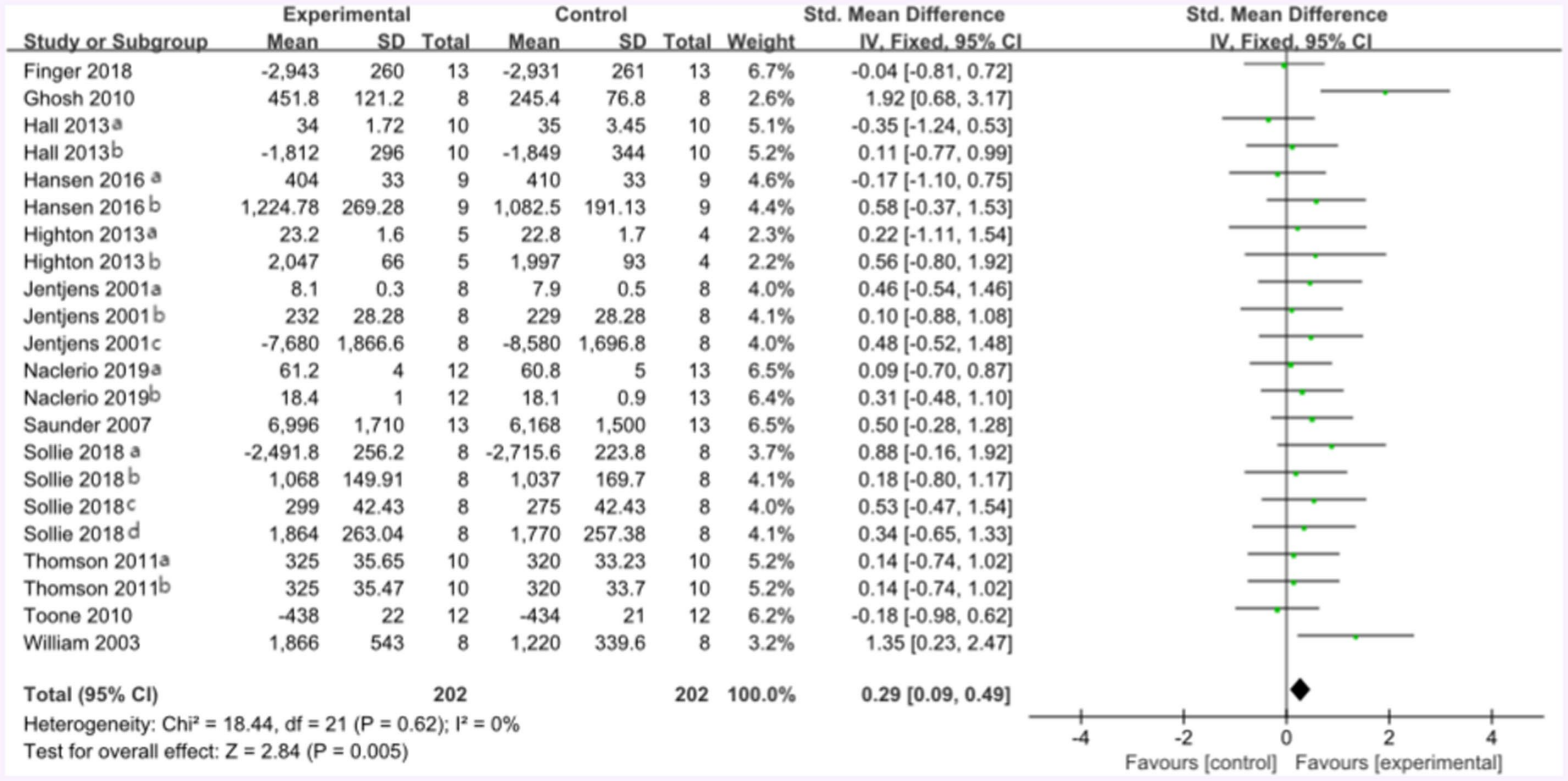
The forest plot of endurance performance after protein plus carbohydrate intervention (CHO group vs. PRO plus CHO group).

The forest plot of endurance performance after protein plus carbohydrate intervention included twelve studies and 142 athletes. The mean effect size was 0.29 with a 95% confidence interval of 0.09 to 0.49, *p* = 0.005, Z = 2.84, I^2^ = 0%. The protein group showed a greater improvement in endurance performance. Figure 11 provides the details.

**Figure 12:**
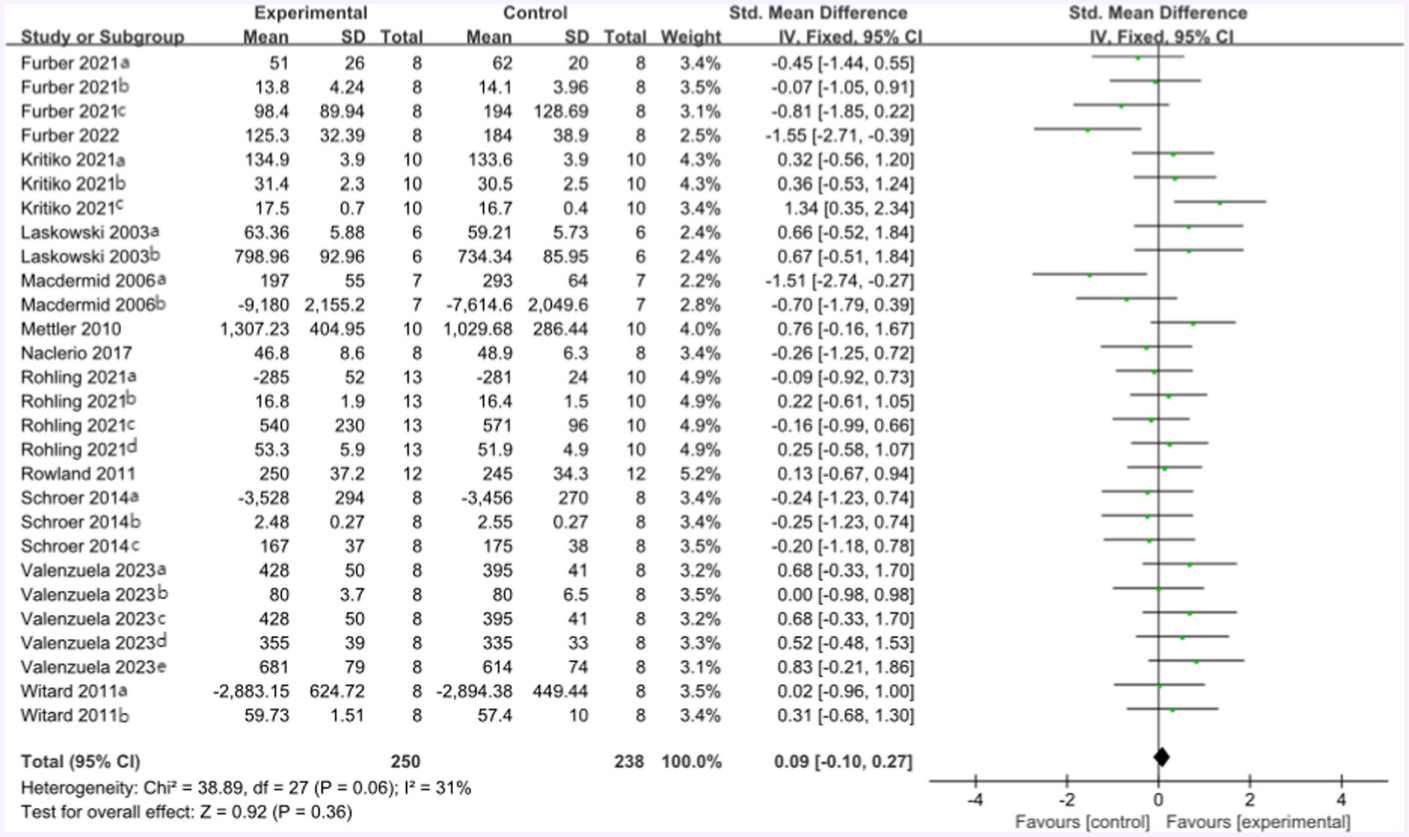
The forest plot of endurance performance after high protein intervention (no protein group vs. high protein group).

The forest plot of endurance performance after the high-protein intervention included 12 studies and 164 athletes. The mean effect size was 0.09 with a 95% confidence interval of −0.10 to 0.27, *p* = 0.36, Z = 0.92, and I^2^ = 31%. No difference between the protein and control groups was observed. Figure 12 provides the details.

**Figure 13:**
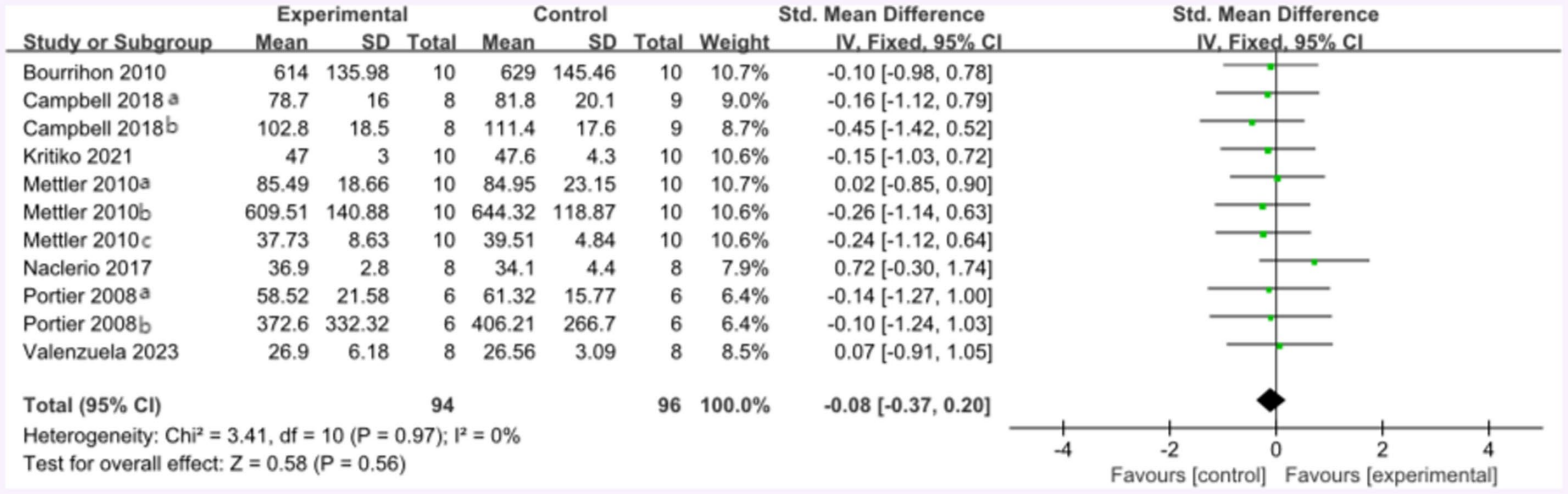
The forest plot of muscle strength after high protein ingestion (no protein group vs. high protein group).

Following the high-protein intervention, the forest plot for muscle strength included seven studies and 117 athletes. The mean effect size was −0.08 with a 95% confidence interval of −0.37 to 0.26, *p* = 0.56, Z = 0.58, and I^2^ = 0%. No difference between the protein and control groups was observed. Figure 13 provides the details.

#### 3.5.4 Subgroup analysis based on each athletic performance test and blood parameters

Table 8 displays the subgroup analysis of each athletic performance test and blood parameters. The protein group showed statistical significance in the subgroup analysis of average speed, Wingate test, time to exhaustion (Vo_2max_ ≤ 90%), and muscle glycogen. The results showed a greater gain for the protein group. The subgroup analysis of time to exhaustion (95% Vo_2max_) revealed that the non-protein group had a higher gain. The remaining data did not show statistical significance in both protein and non-protein groups.

**Table 8:** Subgroup analysis based on the types of specific athletic performance test and blood parameters (non-protein group vs. protein group, final value).

Subgroup name	Studies number	Participants number	SMD or MD (95% CI)	*p*	*Z*	I^2^
Endurance performance						
Maximum speed	4	68	0.29 [−0.14, 0.72]	0.19	1.31	0%
Average speed	3	35	0.39 [0.05, 0.73]	0.02	2.24	0%
Wingate test (peak power)	3	50	0.65 [0.16, 1.15]	0.009	2.61	0%
Time to exhaustion (95% Vo_2max_)	2	32	−1.14 [−1.91, −0.37]	0.004	2.9	0%
Time to exhaustion (Vo_2max_ ≤ 90%)	3	29	1.03 [0.46, 1.60]	0.0004	3.53	52%
Vo_2max_	6	116	0.13 [−0.25, 0.51]	0.5	0.68	0%
Cycling completed time	8	74	−0.11 [−0.45, 0.23]	0.53	0.62	0%
Cycling mean power	5	65	−0.30 [−0.7, 0.09]	0.13	2.16	0%
Muscle strength						
Maximum voluntary contraction (MVC)	3	42	−0.1 [−0.59, 0.39]	0.68	0.41	0%
Counter-movement jump (CMJ)	2	34	−0.14 [−0.66, 0.39]	0.61	0.51	0%
Jump height	4	66	−0.19 [−0.63, 0.25]	0.39	0.86	0%
1RM chest press	2	32	0.03 [−0.56, 0.62]	0.93	0.09	0%
1RM Squat	2	29	−0.07 [−0.68, 0.55]	0.83	0.21	0%
Physiological indices						
Blood glucose	14	153	0.11 [−0.14, 0.37]	0.39	0.86	35%
Muscle glycogen	2	16	0.74 [0.02, 1.47]	0.04	2.02	0%
Heart rate	9	89	−0.07 [−0.38, 0.23]	0.64	0.47	0%
Blood lactate	7	87	0.04 [−0.38, 0.30]	0.83	0.22	0%

#### 3.5.5 Subgroup analysis based on the energy matching or not between protein group and non-protein group

Table 9 displays the subgroup analysis based on energy matching. The meta-analysis in both the energy-matching and non-energy-matching groups did not show statistical significance. The result of the subgroup analysis is the same as the meta-analysis, which means the energy mismatch between the control and experimental groups did not affect the result or cause deviation.

**Table 9:** Subgroup analysis based on the energy matching or not between protein group and no protein group.

Subgroup name	Studies number	SMD (95% CI)	*p*	*Z*	I^2^
Energy matching	20	0.11 (−0.03 to 0.26)	0.12	1.55	22%
Non-energy matching	6	0.07 (−0.17 to 0.31)	0.57	0.56	0%

### 3.6 Sensitivity analysis

**Figure 14:**
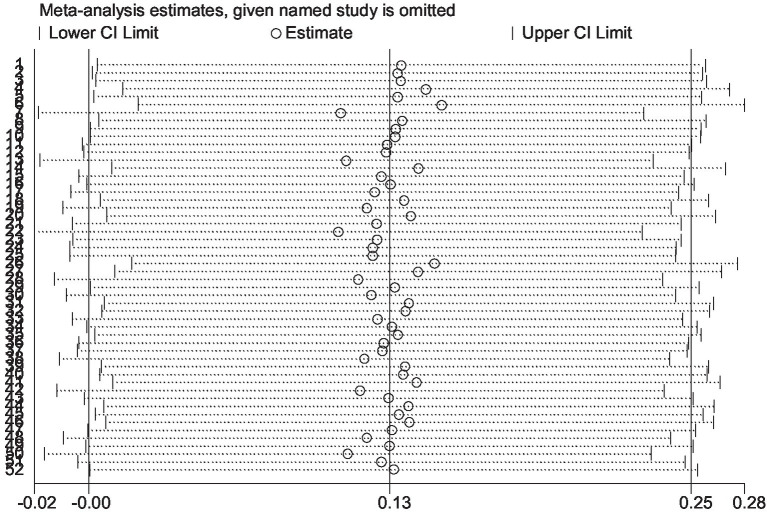
The sensitive analysis of athletic performance.

**Figure 15:**
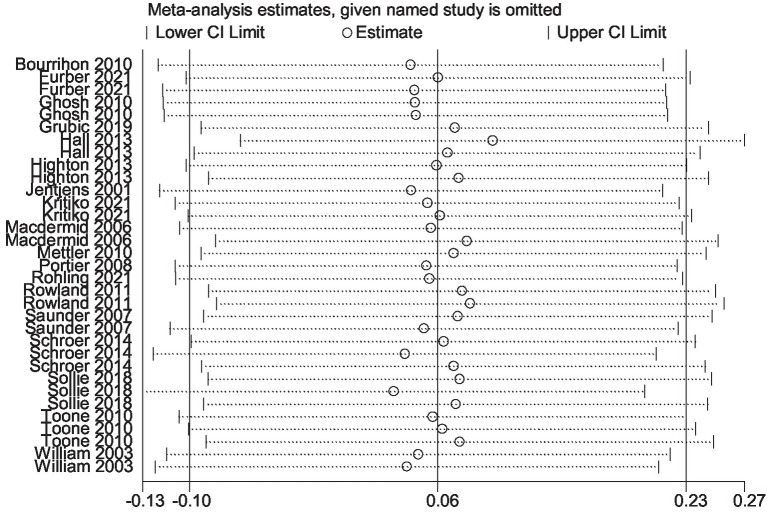
The sensitive analysis of physiological indices.

The results of the sensitive analysis can be seen in Figures 14, 15. In the sensitive athletic performance analysis, this estimated result was 0.126, with a 95% confidential interval of −0.001 to 0.254. The estimated outcome closely matched the results of the meta-analysis on athletic performance. The estimated result in the sensitive analysis of the physiological indices was 0.063, with a 95% confidential interval of −0.099 to 0.226. The estimated result was highly close to the meta-analysis result in the physiological index. After the leave-one-out test, the results remained the same as the meta-analysis result, indicating the excellent robustness of all included data.

### 3.7 The risk of bias

**Figure 16:**
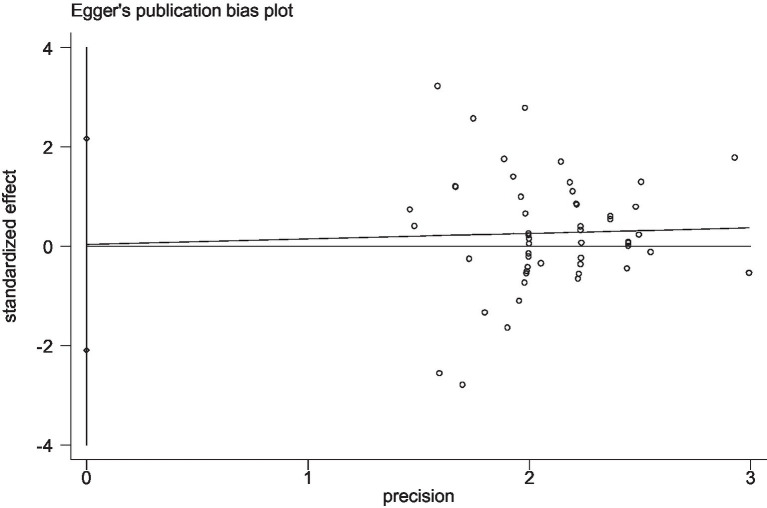
The funnel plot of studies in the athletic performance (Egger’s test).

**Figure 17:**
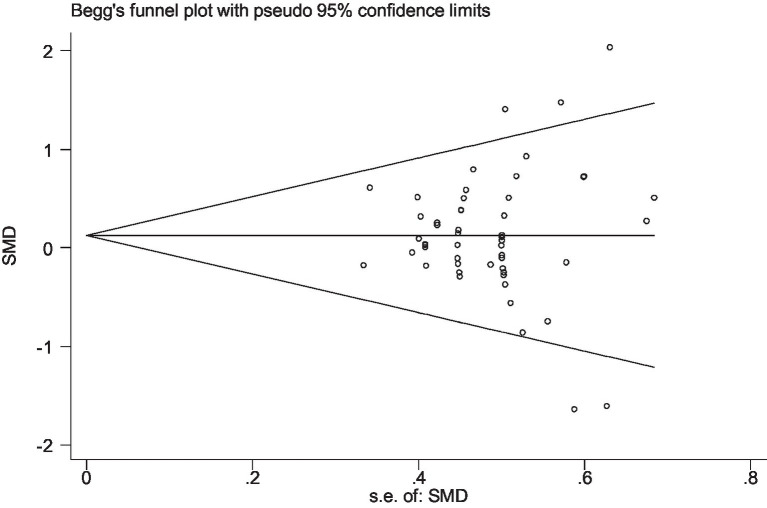
The funnel plot of studies in athletic performance (Begg’s test).

**Figure 18:**
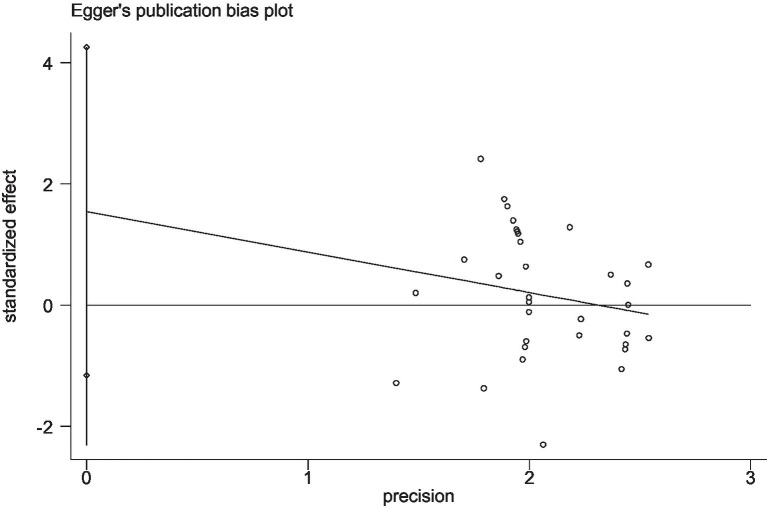
The funnel plot of studies in the physiological index (Egger’s test).

**Figure 19:**
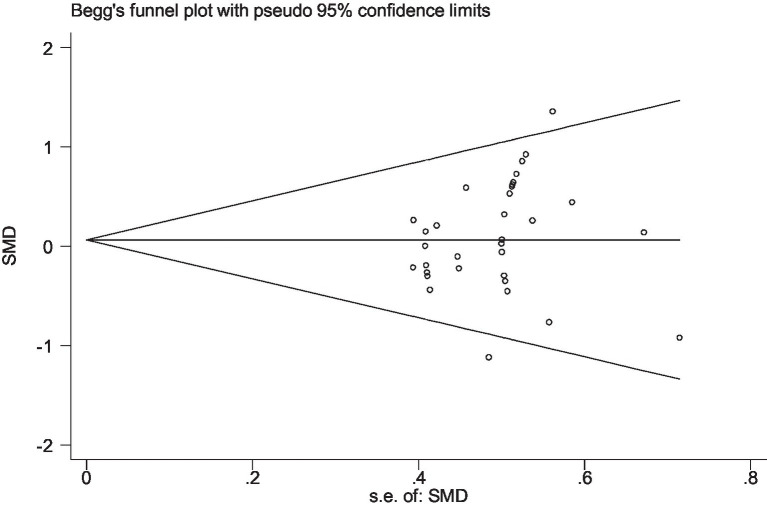
The funnel plot of studies in the physiological index (Begg’s test).

Stata 12 was used to assess the risk of bias in the included studies on athletic performance and physiological indices. Figures 16, 17 summarize the risk of bias in athletic performance studies using Begg’s and Egger’s assessment methods. The Egger assessment result (*p* = 0.826) indicates no significant risk of bias in these studies. Similarly, Figures 18, 19 summarize the risk of bias in studies related to physiological indices, with the Egger assessment result (*p* = 0.301) also indicating no risk of bias in this category. The funnel plot shows symmetrically distributed circle dots, suggesting a low risk of bias but also indicating a study population of potentially low quality.

## 4 Discussion

This is the first meta-analysis to investigate the effect of protein intake, including protein diets and protein supplements, on athletes’ endurance performance, muscle strength, and physiological indices. High protein ingestion could not improve athletic performance, including endurance performance and muscle strength in athletes, but protein co-ingested with carbohydrate supplements was found to have statistical significance through subgroup analysis in improving endurance performance in athletes, especially in anaerobic endurance.

The present systematic review and meta-analysis summarize evidence for the effect of (1) protein intake on muscle strength; (2) protein intake during and/or before the initial bout of exercise on subsequent athletic performance, including aerobic and anaerobic performance and muscle strength; and (3) high protein ingestion versus protein plus carbohydrate co-ingestion on athletic performance in subgroup analysis.

### 4.1 The effect of protein intake on muscle strength

The meta-analysis failed to detect a relationship between protein intake and muscle strength. Many studies have demonstrated that protein intake cannot improve muscle strength in athletes. Protein sources are not likely to have an impact on muscle strength (51, 52), and some researchers found statistical significance between protein intake and lean mass and concluded that lean mass gain may not necessarily translate to strength improvements (51, 53, 54). Schoenfeld et al. concluded that protein timing had a small to moderate effect on muscle hypertrophy, with no significant effect on muscle strength (55). Researchers did not detect a statistically significant effect of protein intake on different age groups except for athletes. A meta-analysis of protein supplements (56) also did not find a statistical significance between the control and protein groups in muscle strength in common people, even including those who suffered from disease and were older. Seven studies’ data were collected to calculate SMD of muscle strength. No correlations between single or combined protein intake and leg muscle power, leg muscle strength, or handgrip strength were observed in older people (57). Regardless of age, it appears that protein intake has no significant impact on muscle strength.

### 4.2 The effect of protein intake on endurance performance

The meta-analysis revealed that protein intake significantly improved endurance performance, including anaerobic and aerobic capacity, as demonstrated by the subgroup analysis of running speed, Wingate test, and time to exhaustion. The effect size in the forest plot of endurance performance presented by the change value was found to have greater statistical significance than the final value. The forest plot of endurance performance presented by the change value was considered the baseline difference among participants, and the results were more convincing than the final value. Many studies had similar results. A review (58) concluded that using protein supplements during recovery can enhance subsequent exercise capacity, particularly when sub-optimal carbohydrate delivery. Lin et al. concluded that protein supplements increased aerobic capacity, stimulated lean mass gain, and improved time trial performance during chronic endurance training in healthy and clinical populations (56). It was also found by Stearn et al. that eating protein and carbs together increased endurance performance when assessed by time to exhaustion and where supplements were matched for CHOs. However, the ergogenic effect of protein observed in studies on isocarbohydrates might be due to the general effect of adding calories (fuel) rather than a specific benefit of protein. Before drawing a clear conclusion, further research is necessary (59).

In the subgroup analysis, protein intake did not show statistical significance in increasing the Vo_2max_ compared to the control group. Protein intake did not provide additional benefits for participants who had already achieved substantial improvement in Vo_2max_ through high-volume training, suggesting that the Vo_2max_ improvement from protein intervention had reached a ceiling effect in these athletes.

However, Lin et al. found statistical significance in Vo_2max_ improvements in the protein group among individuals who were weak or ill, concluding that protein supplements probably provided more benefits to those with lower aerobic capacity but offered few additional benefits to participants who had already achieved substantial improvements in VO_2max_ through high-intensity endurance training (56). Additionally, some researchers discovered that protein supplements increased the production of myofibrillar protein but not mitochondrial protein, indicating that protein intake did not improve whole-body aerobic capacity (i.e., VO_2max_) (60, 61). This finding provides supportive evidence from a molecular perspective.

The time to exhaustion in cycling and running exerted by different loads of Vo_2max_ (95%, 85% & 75%) presented opposing results. The control group experienced a greater increase in the time to exhaustion when running at the load of 95% Vo_2max_, indicating that protein intake was not beneficial in improving anaerobic performance. However, it is important to note that the same author wrote all studies in the forest plot of time to exhaustion in the running and published them in different years. The number of athletes was limited. It still needs further investigation. The time to exhaustion during cycling at 90, 85, and 75% loads of Vo_2max_ exhibited moderate heterogeneity of 52%, which can be attributed to the variation in Vo_2max_ exerted during the test.

Overall, protein ingestion seems to have a beneficial effect on endurance performance, and several studies have given supportive evidence. Ingesting protein during exercise may have an ergonomic effect, potentially delaying the time to exhaustion in tests requiring significant physical strength (62). The provision of protein/amino acids supports increased rates of protein synthesis and positive protein balance following endurance exercise (63). Overall, protein intake has greater benefits for endurance athletes, such as cyclists or runners, than for other types of athletes. Although protein intake may improve endurance performance, more studies are still needed to enhance its credibility.

### 4.3 The effect of protein intake on physiological indices

In the meta-analysis of the physiological indices, we did not find a statistical significance between the protein group and control group, but in the subgroup analysis, the muscle glycogen was found to be statistically significant in the protein group. A sufficient amount of muscle glycogen, increased by protein ingestion, could indirectly improve performance in athletes. A narrative review written by Larsen et al. tried to find the relationship between muscle glycogen and high-intensity exercise, and they concluded that the amount of muscle glycogen was linked to high-intensity tolerance (64). As a result, adequate protein ingestion could improve muscle glycogen content, indirectly increasing athletes’ tolerance for high intensity. The body first consumes muscle glycogen during high-intensity training, followed by blood glucose. Inadequate blood glucose can cause fatigue and hinder athletic performance, while a deficiency in muscle glycogen can have the same effect. Athletes’ endurance performance has a strong correlation with their muscle glycogen stores. An increase in muscle glycogen concentration could indirectly improve endurance performance. Stearns et al. found solid evidence through the marathon match that protein ingestion could save muscle glycogen and blood glucose in the body (59). Manninen et al. concluded that whey protein hydrolysate appeared to enhance the effects of carbohydrate ingestion on post-exercise muscle glycogen resynthesis (65). Thus, protein intake could accelerate the body’s increase in protein oxidation, which in turn reduces the speed at which athletes’ bodies consume sugar and preserves muscle glycogen and blood glucose. The subgroup analysis of muscle glycogen, blood lactate, blood glucose, and heart rate did not reveal a statistically significant difference between the protein and control groups. Furthermore, protein intake did not reduce blood lactate levels following exercise, implying that athletes’ feelings of muscle soreness and fatigue might not alter with sufficient protein intake.

### 4.4 High protein ingestion versus protein plus carbohydrate co-ingestion in athletes

The possibility that the carbohydrate-protein treatment enhances carbohydrate availability has been offered as a potential explanation for enhanced performance or endurance (30, 38, 39). Protein can provide athletes with additional energy, extending their time to exhaustion. The NSCA guide to sport and exercise nutrition mentions that CHOs with protein in a 4:1 ratio before or after prolonged exercise can facilitate greater performance (1). In the subgroup analysis, protein plus carbohydrate co-ingestion found statistical significance in endurance performance, but the high protein ingestion group did not. The benefits of high protein intake were negligible for athletes. Many researchers mentioned the advantage of protein plus carbohydrate co-ingestion. Kloby et al. mentioned that compared with carbohydrate, carbohydrate plus protein co-ingestion appeared to enhance the time to exhaustion (TTE) performance and time trials (TT) performance, and the participants included in their study were healthy adults, both male and female (66). Furthermore, co-ingesting protein with CHOs outperformed carbohydrate ingestion alone, and the subgroup analysis of the Wingate test and TTE revealed that athletes’ endurance performance improved after co-ingesting protein and CHOs compared to the carbohydrate group. Similarly, Stearns et al. concluded that co-ingestion of protein with CHOs during exercise had a benefit (9%) on performance compared to carbohydrate alone, and protein ingestion was not statistically significant in the time-trial studies. In contrast, the time-to-exhaustion studies revealed a significant improvement (59). It has provided robust evidence to support the benefits of protein-carbohydrate co-ingestion.

### 4.5 Limitation

Several limitations must be addressed. First, studies involve different types of proteins, such as soy protein, whey protein, and so on. Plant protein seems to have a different effect on athletic performance than other types of protein. However, due to a lack of studies, we are unable to conduct a meta-analysis based solely on plant protein. We only found two studies that compared the effects of plant protein and whey protein on athletic performance in athletes, and the lack of a comparison group prevents us from incorporating it into our research. It is a new and vague area that deserves more attention. Second, the meta-analysis’s results, derived from several trials with a small sample size of approximately 10 participants in treatment arms, resulted in a low-quality outcome. Therefore, we need high-quality, large-scale studies to validate the current study’s findings. Third, in the subgroup analysis, the samples and studies included in the forest plot were few, like the muscle glycogen. The forest plot of muscle glycogen only included two studies from 2001 and 2003, indicating that while the protein group showed statistical significance, it lacks credibility and requires further studies to bolster its findings in the future. Finally, it appears that when adequate amounts of CHOs are consumed, protein supplements do not further increase aerobic performance (42, 53), and it cannot be investigated through this meta-analysis.

## 5 Conclusion

This systematic review and meta-analysis included 28 RCT studies involving 373 athletes. While the overall meta-analysis of athletic performance did not find statistically significant effects, subgroup analysis showed that protein ingestion had a beneficial effect on endurance performance and muscle glycogen levels, suggesting that athletes may benefit more from co-ingesting protein with CHOs rather than relying solely on high protein intake.

Protein intake appears to support protein oxidation, which helps preserve muscle glycogen and indirectly enhances endurance performance. However, protein ingestion, whether alone or combined with CHOs, did not improve muscle strength in athletes. Additionally, different types of proteins may vary in their effectiveness on athletic performance. Future research should focus on the efficacy of plant protein on athletic performance.

Further studies should incorporate physical tests, particularly focused on measuring muscle glycogen levels. Additionally, future research should ensure the blinding of outcome assessment during the experiment to reduce potential bias. The small sample size of athletes in existing studies has contributed to low-quality outcomes, highlighting the need for larger, well-designed studies to provide more robust and convincing evidence to support these findings.

## Data Availability

The original contributions presented in the study are included in the article/supplementary material, further inquiries can be directed to the first author.
